# Electricity in Modern Medicine

**Published:** 1911-11-11

**Authors:** Alfred C. Norman

**Affiliations:** House Surgeon, Sunderland and Durham County Eye Infirmary, and late House Surgeon in charge of X-Ray Department, Royal Buckinghamshire Hospital.


### ELECTRICITY IN MODERN MEDICINE.

By ALFRED C. NORMAN, M.D. Edin., House Surgeon, Sunderland and Durham County Eye Infirmary, and late House Surgeon in charge of X-Ray Department, Royal Buckinghamshire Hospital.

In the days when the application of electricity to the industrial arts was in its infancy, when electricity was regarded by many as being almost akin to witchcraft, and when even its most ardent exponents were not always certain what it might do next, there were a few enthusiasts who prophesied a great future for it in medicine. Since those days its study has become an exact science, which has revolutionised many branches of medical diagnosis and has made itself almost indispensable in the treatment of certain diseases.

Thanks to electricity and the invention of the metallic-filament lamp, there is now practically no cavity of the living body which we cannot explore. The cystoscope has become an instrument of routine application, and following in its footsteps we have the urethroscope, the rectoscope, the sigmoidoscope, the pharyngoscope, the direct-vision laryngoscope, the tracheo-bronchoscope, the œsophagoscope, and, perhaps most wonderful of all, the gastroscope, not to mention the various transilluminators for the frontal sinuses and the antrum of Highmore.

In ophthalmology the introduction of the Marple illuminated ophthalmoscope has very materially simplified direct examination of the fundus oculi.

The *x*-ray diagnosis of fractures and dislocations and of many diseases of the bones and joints has now become a commonplace in modern medicine, while our knowledge of the functions and pathology of the more inaccessible organs, such as the stomach and intestines, the kidneys and ureters, and even the heart, the aorta and the apices of the lungs, has been, to say the least of it, very materially broadened by the advent of the *x*-ray. The almost inestimable value of *x*-ray localisation of foreign bodies in the eye and other tissues can only be fully appreciated by those who practise in districts where accidents to workmen are of everyday occurrence.

When we come to the question of treatment we are compelled to recognise the great value of electricity in widening the scope of our therapeutic armamentarium. Galvanism and Faradism, static electricity and high-frequency currents; light baths, ionic medication and the *x*-rays all play their part in a well-equipped electrical department, to lighten the burden of suffering humanity and hasten the cure of many previously intractable diseases.

In short, no modern hospital with any pretensions to being up to date can afford to be without so essential an aid to diagnosis and treatment as an *x*-ray and electrical department. Money spent in acquiring and adequately furnishing such a department is money well spent indeed. If the committees of the smaller hospitals in the provinces would only realise how much could be done by the judicious expenditure of £100 on electrical equipment the efficiency of many of these hospitals would be enormously increased.

Within the scope of this article will be discussed the various electrical instruments used in medicine, their cost, the principles governing their use, and the fitting up of *x*-ray and electrical departments suited to hospitals of limited size and means.

Before going into the practical side of the subject it might be well to consider here a few of those elementary principles in electricity which, if thoroughly grasped, will go far to bring about that perfect understanding between operator and instrument which makes for the production of constant and uniform results. If the principles about to be enunciated are kept well in mind, a working knowledge of medical electricity is very easily acquired; and yet how often do we see good instruments spoilt or laid on one side as useless, or even blamed for bad results, when the fault really lies in the ignorance of the operator who has never taken the trouble to acquire the few fundamental facts necessary to their understanding.

To begin at the beginning, there are three factors which have to be considered in utilising an electric current—viz., Ampèrage, Voltage, and Resistance.

*Ampèrage* is the amount or quantity of current passing through any point in a circuit at a given moment. It is measured by means of an ampère-meter.

*Voltage*, also known as electromotive force, is the pressure at which electricity is discharged from its source. It may be measured with a volt-meter. Voltage may be regarded as the factor which overcomes the resistance of the circuit. It does no work *per se*, but, so to speak, it forces the ampères through the circuit.

*Resistance.*—The resistance of a conductor to a current of electricity depends upon its conductivity (an inherent property), its section, and its length. Some metals are worse conductors than others, and have therefore a greater resistance. A thin wire has more resistance than a thick one of the same metal, a long wire more than a short one. The unit of resistance is one ohm.

Let us now apply these facts to a simple source of electricity, such as an ordinary storage battery or accumulator. The simplest form of accumulator consists of a single cell containing positive and negative plates, which when fully charged can discharge electricity at a pressure of about two volts. Whether the cell be made as small as a thimble or as big as a bucket the current will always be given off at a pressure of about two volts, but the larger cell will be capable of discharging a certain number of ampères for a longer time than the small one before becoming exhausted, just as a large reservoir could discharge so many gallons of water over a longer period than a small one. The capacity of an accumulator is usually designated in ampère-hours; a thirty ampère-hour cell would discharge 1 ampère for 30 hours, or 2 ampères for 15 hours, or ½ ampère for 60 hours before requiring to be re-charged.

How will such a cell behave under the various conditions of work required of it? The pressure is constant (2 volts); the output at a given time must depend upon the resistance in the circuit. As long as the positive and negative terminals of the cell are not connected by a conductor no current will flow between them, because the resistance of air is too great to permit of a current of 2 volts jumping across the air-gap which separates the terminals. Suppose we first complete the circuit by connecting the terminals with a coil of fine platinoid wire; this has a very high resistance, and we should get but a small fraction of an ampère flowing through it. Let us next connect the terminals of our cell to the two ends of a cautery burner; this has a comparatively low resistance, and we should consequently get a much greater current through the circuit—16 ampères perhaps—which would exhaust our cell in less than two hours. Lastly, suppose we short-circuit the cell—*i.e.* connect the terminals with a thick piece of metal whose resistance is negligible—we should have a sudden flow of tremendous ampèrage which would exhaust the cell in a few seconds and, incidentally, ruin the plates. We may conclude therefore that the ampèrage flowing through a circuit at a given moment depends upon the resistance of that circuit when the voltage is constant.

Let us now see what happens if the resistance remains constant while the voltage is increased. By connecting two cells together in series we can make a battery of 4 volts; three cells will form a 6-volt battery, and so on indefinitely. Now suppose we take a piece of resistance wire of such length and thickness that would allow 1 ampère to pass when connected for a moment to the terminals of a 2-volt cell, this same wire would allow 2 ampères to pass when connected to a 4-volt battery, or 3 ampères when connected to a 6-volt battery. Thus we see that when the resistance is constant the ampèrage flowing at any moment through a circuit depends upon the voltage of the supply.

To sum up the last two paragraphs, we may say that the amount of current flowing through a circuit at a given moment is dependent upon the voltage of the supply and the resistance of the circuit. Now this is nothing more than Ohm's law. In our student days we saw it expressed by the formula $C = \frac{E}{R}$, which being interpreted means that the current in ampères (C) varies directly as the electromotive force in volts (E), and inversely as the resistance in ohms (R).

If the voltage of the supply be known and the ampèrage flowing at the moment be read off from the meter, it is easy to calculate the total resistance in the circuit. Obviously in the illustration just given the resistance of the piece of wire must have been 2 ohms, for the battery of 4 volts gave a current of 2 ampères through it, and by transposing Ohm's law we know that $R = \frac{E}{C}$, but $E=4$ volts and $C=2$ ampères, therefore R must have been $\frac{4}{2} = 2$ ohms.

This is all the theory we need discuss, but it will be constantly referred to in the subsequent sections.

(*To be continued.*)

